# Microcystic Variant of Urothelial Carcinoma

**DOI:** 10.1155/2013/654751

**Published:** 2013-12-02

**Authors:** Anthony Kodzo-Grey Venyo

**Affiliations:** North Manchester General Hospital, Department of Urology, Delaunays Road, Manchester, UK

## Abstract

*Background*. Microcystic variant of urothelial carcinoma is one of the new variants of urothelial carcinoma that was added to the WHO classification in 2004. *Aims.* To review the literature on microcystic variant of urothelial carcinoma. *Methods.* Various internet search engines were used to identify reported cases of the tumour. *Results*. Microscopic features of the tumour include: (i) Conspicuous intracellular and intercellular lumina/microcysts encompassed by malignant urothelial or squamous cells. (ii) The lumina are usually empty; may contain granular eosinophilic debris, mucin, or necrotic cells. (iii) The cysts may be variable in size; round, or oval, up to 2 mm; lined by urothelium which are either flattened cells or low columnar cells however, they do not contain colonic epithelium or goblet cells; are infiltrative; invade the muscularis propria; mimic cystitis cystica and cystitis glandularis; occasionally exhibit neuroendocrine differentiation. (iv) Elongated and irregular branching spaces are usually seen. About 17 cases of the tumour have been reported with only 2 patients who have survived. The tumour tends to be of high-grade and high-stage. There is no consensus opinion on the best option of treatment of the tumour. *Conclusions*. It would prove difficult at the moment to be dogmatic regarding its prognosis but it is a highly aggressive tumour. New cases of the tumour should be reported in order to document its biological behaviour.

## 1. Introduction

Since microcystic variant of urothelial carcinoma was added to the WHO classification, very few cases have been reported in the literature, and in view of this most practitioners would be unfamiliar with the biological behaviour of this rare type of tumour. The ensuing paper contains literature review of microcystic variant of urothelial carcinoma.

## 2. Methods

Various internet search engines including Google, Google Scholar, PubMed, Educus. and UpToDate were used to identify the literature including case reports, case series, and review papers on microcystic variant of urothelial carcinoma for the review of the literature on microcystic variant of urothelial carcinoma. The key words that were used included microcystic variant of urothelial carcinoma and microcystic transitional cell carcinoma. In all 17 documentations were found which were relevant to the aetiology, presentation, diagnosis, management, and outcome of microcystic variant of urothelial carcinoma.

## 3. Literature Review

### 3.1. Overview

#### 3.1.1. Definition and Terminology

Microcystic variant of urothelial carcinoma is one of the variants of urothelial carcinoma that was added to the WHO classification in 2004. Microcystic variant of urothelial carcinoma has predominant features of urothelial carcinoma. However, it has prominent inter- or intracellular lumina and no true glands. Microcystic variant of urothelial has been considered to be equivalent to urothelial carcinoma with gland-like lumens by AFIP authors [1]; nevertheless, the World Health Organization has made a minor distinction between microcystic variant of urothelial carcinoma and urothelial carcinoma with gland-like lumina [2]. Aetiology

Postulates that have been put forward regarding the aetiology of the microcystic variant of urothelial carcinoma include the following.

- The cyst-like structures may emanate from the ability of urothelium to form and line spaces as occurs in the urinary bladder.
- The cyst-like structures may result from cell degeneration which is based upon presence of luminal debris and necrotic cells [3].

#### 3.1.2. Characteristic Peculiarities of Microcystic Variant of Urothelial Carcinoma

Paz and associates [4] described the clinical and histological features of 12 transitional cell carcinomas of the urinary bladder with microcysts and concluded that microcystic variant of transitional cell carcinoma was linked with high-stage and high-grade urinary bladder tumours and with other primary tumours, especially those of the colon. They suggested screening these patients for asymptomatic tumours of the colon.

#### 3.1.3. Microscopic Peculiarities of Microcystic Urothelial Carcinoma Variant

Microscopic features of microcystic variant of urothelial carcinoma listed by Rugvedita [3] include the following.

- Prominent intracellular or intercellular lumina/microcysts which are surrounded by neoplastic urothelial or squamous cells.
- The lumina tend to be empty, but they may contain granular eosinophilic debris, necrotic cells, or mucin.
- The cysts may be variable in size; they may be oval or round; they may be up to 2 mm; they are lined by urothelium. The cells are flattened cells or low columnar cells; however, they are not colonic epithelium or goblet cells.
- The cysts tend to be infiltrative and they may invade the muscularis propria.
- The pattern of the tumour on microscopic examination mimics cystitis glandularis cystica.
- Elongated, irregular branching spaces can be seen on microscopic examination.

Young and Zukerberg [5] in 1991 observed that four transitional cell carcinomas of the urinary bladder on microscopic examination had cysts.

#### 3.1.4. Immunopathology Including Immunohistochemistry

Based upon the report of Pacchioni and associates [6] it would be suggested that on rare occasions microcystic variant of urothelial carcinoma may have neuroendocrine differentiation.

Some of the immunohistochemistry characteristics of microcystic variant of urothelial carcinoma include

- positive staining with 34*β*E12, p63, CK7, uroplakin, and thrombomodulin,
- negative staining with *α*-methyl-coenzyme A racemase,
- Ki-67 and p53 overexpression in high-grade cancers [7].

#### 3.1.5. Differential Diagnoses

Some of the conditions that may mimic microcystic urothelial carcinoma include the nested variant of urothelial carcinoma; urothelial carcinoma with glandular differentiation; adenocarcinoma; cystitis cystica and cystitis glandularis; mullerianosis; nephrogenic adenoma and nephrogenic metaplasia; and mucoid cytoplasmic inclusions.

The nested variant of urothelial carcinoma may exhibit focal tubular differentiation. Urothelial carcinoma with glandular differentiation may look similar to microcystic variant of urothelial carcinoma; however, it has true glands which are lined by goblet cells or colonic epithelium, and it is primarily a urothelial tumour in contrast to adenocarcinoma [3]. Nephrogenic metaplasia/adenoma are small hollow tubules which are similar to mesonephric tubules, and these tend to be lined by a single layer of bland cuboidal or hobnail cells, surrounding eosinophils or basophilic secretions, which are typically not cystic; they either do not have atypia or there is minimal atypia or mitotic figures. There is also no true invasion [3]. It has been stated that in mullerianosis there is presence of 2 of 3 of endocervicosis, endosalpingiosis, or endometriosis and no mitotic figures or atypia [3].

Rugvedita [3] stated that mucoid cytoplasmic inclusions may be present in up to 37% of urothelial carcinomas, often of high grade. Rugvedita [3] stated that adenocarcinoma tends to exhibit diffusely lined goblet cells or intestinal cells and not flattened urothelial-like cells and that they are usually deeply invasive and of high grade. Periodic acid-Schiff-positive cytoplasmic inclusions were also found by Donhuijsen et al. [8] in some microcystic variant of urothelial carcinoma.

#### 3.1.6. Outcome

With regard to outcome of microcystic variant of urothelial carcinoma, Barresi and associates [9] stated that microcystic urothelial carcinoma is a rare variant of transitional cell carcinoma with an indefinite prognostic significance.

### 3.2. Miscellaneous Narrations from Reported Cases (Table 1)

Paz and associates [4] stated that among 940 patients with transitional cell carcinoma of bladder diagnosed in their institution during a 5-year period, 12 (1.2%, eight men and four women, mean age 71.1 years, range 52 years to 85 years) were diagnosed histologically as having microcystic transitional cell carcinoma; out of these three had tumours that were confined to the epithelium, six had tumour invasion of the lamina propria, and three had muscle-invasive tumours. One patient had low-grade transitional cell carcinoma and 11 had high-grade transitional cell carcinoma. Six patients had a second primary tumour, three had carcinoma of colon, one had a villous adenoma of the caecum, one had a locally advanced carcinoma of the prostate gland, and the last patient had a squamous cell carcinoma of the uterine cervix [4].

Young and Zukerberg [5] reported four transitional cell carcinomas of the urinary bladder which on microscopic examination were found to have cysts. They reported that the tumours occurred in patients from 35 years to 69 years of age and that the tumours were deeply invasive, two were of grade 2, and two were of grade 3 of 3 grades. Young and Zukerberg [5] stated that the cysts were prominent in all the cases and predominated in one of them. In the latter case the cysts were the cause of major problems in interpretation. Young and Zukerberg [5] additionally reported that the cysts, which usually were round to oval, were as large as 1.2 mm and were of varying sizes. They were usually lined by transitional cells or low columnar cells showing mucinous differentiation; occasionally they were lined by a single layer of flattened cells or the lining epithelium was denuded. Elongated, irregular branching spaces were also found. Young and Zukerberg [5] iterated that recognition of these tumours is facilitated by their association with typical transitional cell carcinoma, but when the cysts predominate, awareness of this change may be essential in the establishment of the diagnosis, especially in a biopsy specimen [5].

Pacchioni and associates [6] reported a case of microcystic urothelial cell carcinoma arising in the renal pelvis and showing focal neuroendocrine differentiation. They reported a 55-year-old man with a history of non-small-cell carcinoma of the lung who presented with abdominal pain and haematuria. He had imaging studies which revealed a partially cystic mass in the left kidney. Microscopic examination of the nephrectomy specimen revealed invasive carcinoma with prominent microcystic features, with microcysts lined by low columnar and flat cells. Immunohistochemical analysis confirmed the urothelial histotype (positive for thrombomodulin, p63, and high-molecular-weight cytokeratins) and disclosed focal neuroendocrine differentiation [6].

Donhuijsen and associates [8] undertook a histological analysis of 100 cases of urothelial carcinoma. They reported that, overall, 37 cases revealed periodic acid-Schiff-positive cytoplasmic inclusions. These were observed in 14% of grade 1, 49% of grade 2, and 63% of grade 3 carcinomas. The inclusions were histochemically, immunohistochemically, and ultrastructurally identified as cytoplasmic deposits of mucoid materials. Two types of deposits, condensed and noncondensed, could be distinguished. They stated that the demonstration of mucoid deposits in otherwise poorly differentiated metastatic carcinomas may be of some differential diagnostic importance in so far as urothelial carcinoma has to be considered as the possible primary tumour [8].

Barresi et al. [9] reported the acquisition of microcystic histology in the penile metastasis of a high-grade urothelial carcinoma of the urinary bladder. The patient died of disseminated disease six months later. They stated that the immunohistochemical evaluation of mucin expression in the primitive and metastatic tumour suggested that the microcystic histotype may descend from the primitive urothelial carcinoma through a process of dedifferentiation and subsequent redifferentiation. Barresi and associates [9] concluded that the acquisition of microcystic histology seemed to be associated with aggressive clinical course of the urothelial carcinoma, as already suggested by other authors. They suggested that further studies investigating mucin expression in microcystic urothelial carcinoma may help in defining the histogenesis of the tumour [9].

Alvarado-Cabrero and associates [10] presented the case of an 80-year-old man with superficial papillary urothelial carcinoma of the urinary bladder with striking multicystic architecture with a combination of features of urothelial carcinoma with gland-like lumina, with signet-ring cell differentiation and microcystic pattern. However, the tumour shared the morphologic features of several variants of urothelial carcinoma, and the most important differential diagnosis covered the so-called florid Brunneriosis, cystitis cystica, and primary adenocarcinomas of the urinary bladder.

Leroy and associates [11] stated that urothelial carcinoma can present many variable features, which can pose diagnostic difficulties, and that some variants mimicking benign lesions such as cystitis cystica or nephrogenic metaplasia have been described as deceptively bland bladder carcinoma. Leroy and associates [11] reported 2 cases of microcystic variant of urothelial carcinoma that developed in the pelvicalyceal system as follows.

**Case 1**

A 73-year-old man had a 5-year history of superficial transitional cell carcinoma of the urinary bladder. He had presented with visible haematuria. He underwent cystoscopy which did not reveal any abnormality in the urinary bladder; however, there was blood exuding from the right ureteric orifice. He underwent computed tomography scan and urography which did not show any tumour. A right nephrectomy was performed because of recurrent haematuria with anaemia. He died of his disease 18 months later with pulmonary metastases which was proven by transthoracic fine-needle biopsy.

**Case 2**

A 62-year-old woman presented with visible haematuria of a few weeks duration. She underwent cystoscopy and bladder biopsies and the histology was normal. Cytological examination of her urine revealed atypical urothelial cells. She had computed tomography scan and urography which revealed a tumour in the upper calyx of the left kidney. She underwent a left nephroureterectomy. She had remained well without evidence of recurrence 6 months after presentation.

Leroy and associates [11] reported the following.

**Figure 1:**
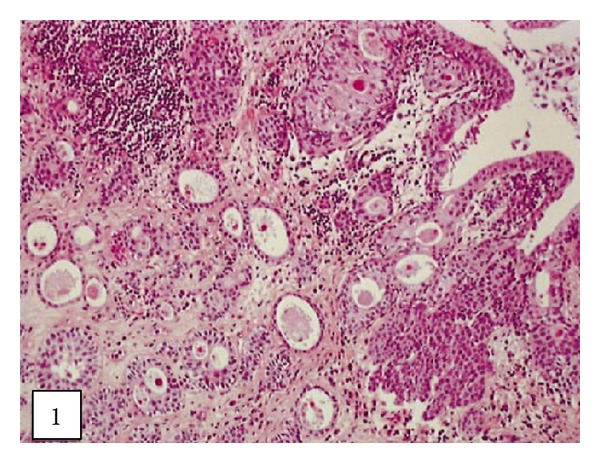
Cysts of various sizes infiltrating the pyelocaliceal cavities (Case 1) (hematoxylin-eosin-saffron, original magnification 3100). Taken from [11] reproduced with permission of the Editor-in-Chief of Archives of Pathology and Laboratory Medicine on behalf of the editorial board of the journal.

**Figure 2:**
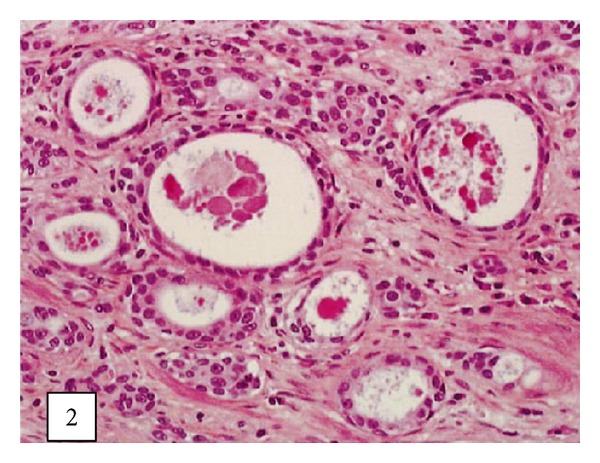
Neoplastic cystic structures with bland appearance invading the renal cavities (Case 1) (hematoxylin-eosin-saffron, original magnification 3200). Taken from [11] reproduced with permission of the Editor-in-Chief of Archives of Pathology and Laboratory Medicine on behalf of the editorial board of the journal.

**Figure 3:**
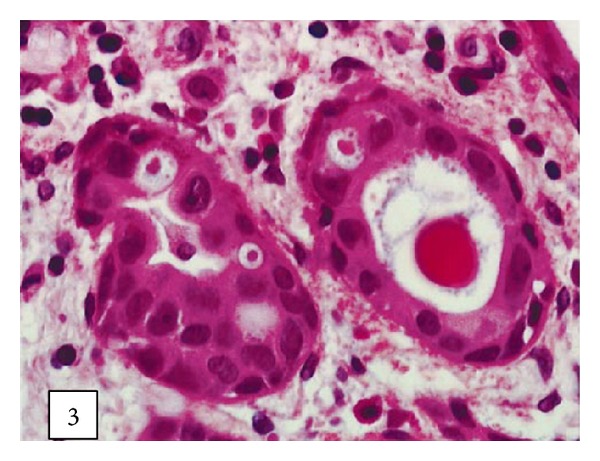
The cysts are lined by cuboidal or flattened cells with minimal cytological atypia (Case 1) (hematoxylin-eosin-saffron, original magnification 3400). Taken from [11] reproduced with permission of the Editor-in-Chief of Archives of Pathology and Laboratory Medicine on behalf of the editorial board of the journal.

**Figure 4:**
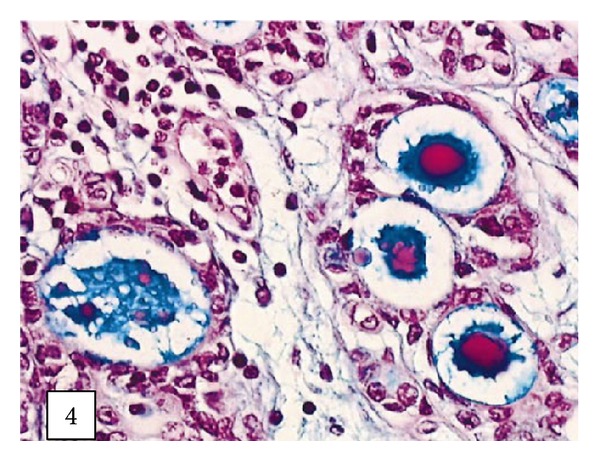
Blue and eosinophilic secretions in the lumens of cysts (Case 1) (Alcian blue/periodic acid-Schiff, original magnification 3400). Taken from [11] reproduced with permission of the Editor-in-Chief of Archives of Pathology and Laboratory Medicine on behalf of the editorial board of the journal.

**Figure 5:**
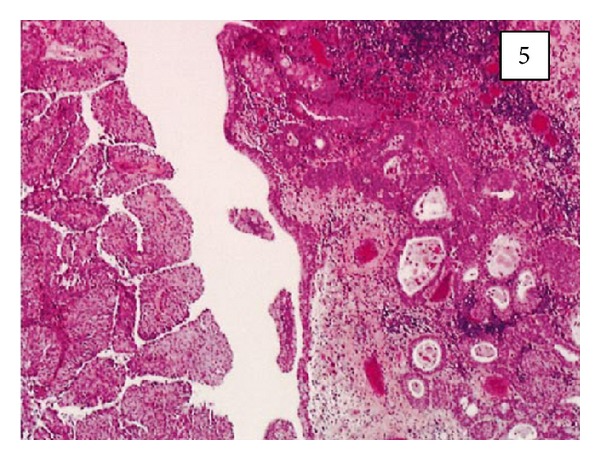
Low-grade papillary urothelial carcinoma associated with an invasive cystic component (Case 2) (original magnification 3100). Taken from [11] reproduced with permission of the Editor-in-Chief of Archives of Pathology and Laboratory Medicine on behalf of the editorial board of the journal.

- Macroscopic examination of the nephrectomy specimen in Case 1 revealed an ulcerated and ill-defined lesion in the renal pelvis extending to the renal parenchyma. Macroscopic examination in Case 2 revealed a friable exophytic tumour of the upper calyx with extension into the renal parenchyma.
- Microscopic examination in the first case revealed a proliferation of small tubules and numerous cysts in the lamina propria and muscularis propria (Figures 1 and 2) and in the renal parenchyma. The cystic structures involved the fat of the renal pelvis and there was no associated papillary carcinoma. The tubules and cysts were randomly distributed with a deeply invasive arrangement in the stroma between muscular fibres and renal tubules. The cystic structures were varied in shape and size; however, they always measured less than 2 mm. Small, cuboidal, or flat cells which were arranged in 1 to several layers lined the cysts and tubules (Figure 3). The tumour cells had an eosinophilic cytoplasm with round and chromatic nuclei. Small nucleoli were also seen. The mitotic activity of the tumour was low (1-2 mitotic figures per 10 high-power fields). There was no evidence of necrosis. Within the lumen of the cysts, eosinophilic or blue secretions were frequently observed, with at times targetoid secretion. These secretions stained positively with Alcian blue and periodic acid-Schiff (Figure 4). Microscopic examination in the second case revealed a papillary urothelial carcinoma of low grade and this was seen to be associated with an infiltration of small tubules and cysts with a deceptively bland appearance (Figure 5). The cystic spaces were at times large, irregular, and elongated. The tumour cells were cuboidal and they had minimal cytological atypia. The mitotic activity of the tumour was low, without abnormal mitosis. There was no evidence of clear or hobnail cells. Rare nests of typical neoplastic transitional cells were found focally [11].

Vardar and associates [12] reported a 52-year-old man with microcystic urothelial carcinoma in the urinary bladder without significant medical history. He underwent transurethral resection of bladder tumour, and histological examination of the specimen was consistent with microcystic variant of urothelial carcinoma. Microscopic examination revealed a muscle-invasive transitional cell carcinoma with prominent cystic features. The cysts were lined by multiple layers of flattened or cuboidal cells with atypia and they were of varying sizes. Immunohistochemical analysis using the streptavidin-biotin peroxidase technique and immunoreactivity for different cytokeratins was performed which confirmed the pathologic diagnosis. He underwent radical cystectomy and pelvic lymphadenectomy. At 10-month followup, the patient did not have any recurrence [12].

Sari and associates [13] reported a 56-year-old man who presented earlier to an outside institution with visible haematuria of one-year duration. He had ultrasound scan which revealed three solid masses on the right and posterolateral walls of the urinary bladder. He did not return until 6 months later. He underwent transurethral resection of the urinary bladder mass. Histological examination of the specimen was reported as grade 3 urothelial carcinoma infiltrating the lamina propria (G3pT1). He next had six intravesical instillations of Bacille Calmette Guerin (BCG). He then had a computed tomography scan which showed a mass extending all the way through the posterior wall of the bladder to the base of the bladder and to the extra-vesical region. A second transurethral resection of bladder tumour was undertaken and histological examination of the specimen revealed that the tumour had infiltrated the muscularis propria. He was next referred to the institution where Sari and associates [13] worked. During admission to the second hospital, he developed visible haematuria and pain in his penis. On examination, his glans penis was found to be hyperaemic and ulcerated, and painful fibrotic tissues were palpated on both corpora cavernosa. His pain and penile lesions persisted despite the use of antibiotics. He had tru-cut biopsy of the penis. Histological examination of the specimen showed that the lesion was a urothelial carcinoma. The patient's pain was intolerable. A radical cystoprostatectomy and total penectomy were performed. Macroscopic examination of the specimen revealed that the tumour was located mainly in the trigone of the urinary bladder, but the prostate gland and the whole body of the glans of the penis were widely infiltrated. Microscopic examination revealed that the tumour was high-grade urothelial carcinoma, of which the invasive part consisted entirely of variable sized cysts which were lined by single to several layers of urothelial cells or rarely by squamous cells. Sari and associates [13] reported that histological examination also revealed that the centres of the cysts were quite often filled with either pale-pink to eosinophilic secretion or necrotic material which stained positive for mucin (Alcian blue). Urothelial cells at the periphery of the many large cysts were punctuated by many smaller cysts that contained a targetoid secretion which was eosinophilic. Sari and associates [13] stated that immunohistochemical staining of the tumour revealed that the tumour cells exhibited diffuse intense staining for CK7 but negative staining for PSA; the percentages of positive staining for Ki-67 and p53 were 15% and 70%, respectively. The tumour infiltrated extensively the muscularis propria, penetrated into the perivesical fat tissue, and extended through the entire penis inclusive of the body, corpus spongiosum, and glans penis. There was evidence of metastasis in one out of 5 obturator nodes removed. The patient next received chemotherapy which included gemcitabine and cisplatinum. There was no evidence of metastatic spread elsewhere. In the first metastatic workup there was no evidence of distant metastasis. However, 3 months later computed tomography scan revealed multiple metastases to the lung and liver. He died of disseminated disease 6 months pursuant to the operation.

Sari and associates [13] iterated the following.

- Up to 2007, 17 cases of microcystic variant of urothelial carcinoma had been reported in the literature.
- The characteristic features of microcystic variant of urothelial carcinoma were first comprehensively described on four cases by Young and Zukerberg [5].
- Twelve of the other cases which were published in the literature by Paz and associates [4] in the earlier years were debated by Leroy and associates [11] in their report of microcystic variant of urothelial carcinoma in the renal pelvis.
- Leroy and associates [11] were of the opinion that the microphotographs presented in the study of Rugvedita [3] were papillary urothelial carcinoma with glandular metaplasia rather than microcystic carcinoma which was described by Young and Zukerberg [5].
- There was no information regarding Ki-67 and p53 reactivity in microcystic variant of urothelial carcinoma. The neoplastic cells in their case exhibited high Ki-67 and p53 expression (15% and 70%, resp.).
- Even though there was a need for a larger number of cases in order to determine the immunohistochemical profile of microcystic urothelial carcinoma, given the high Ki-67 and p53 proteins could be of help in diagnosing malignancy in small biopsy specimens.

Young and associates [5, 14] stated that the differential diagnosis of microcystic variant of urothelial carcinoma with adenocarcinoma is mainly based upon the nature of the cells comprising the cyst wall; glandular cells are seen in adenocarcinoma versus urothelial cells in microcystic variant of urothelial carcinoma [14].

Sari and associates [13] stated that according to the limited number of studies available, microcystic urothelial carcinoma has invasive behavior, and that data on survival rates of these patients from three studies demonstrated that only two patients, both of whom had muscle-invasive tumours, were reported to be alive 3 and 6 years after the diagnosis [5, 15].

Radopoulos and associates [15] reported a 38-year-old man who had microcystic transitional cell carcinoma involving the urinary bladder. The tumour was muscle-invasive and a radical cystectomy was performed. The patient did not receive postoperative chemotherapy or radiotherapy. He did not have any signs of local recurrence or distant metastasis after 3 years of intense followup. Radopoulos and associates [15] stated that even though the number of cases of microcystic variant of urothelial carcinoma documented is so far not sufficient to draw conclusions regarding its optimal treatment, their case would indicate that aggressive therapy is associated with good control of the disease locally and distally.

#### 3.2.1. Penile Metastasis

Pomara and associates [16] stated that despite abundant blood supply, tumours metastatic to the penis are not common, with approximately 300 cases reported since 1970. Demuren and Koriech [17] stated that bladder carcinomas only very rarely metastasize to the penis; nevertheless, 75% of the cases with penile metastases originate from genitourinary organs, with urinary bladder being the most frequent site, and this accounts for 32% of cases.

## 4. Conclusions

Microcystic variant of urothelial carcinoma is a rare tumour and to the knowledge of the author less than 20 cases have been reported in the literature.

In view of the limited number of reported cases of microcystic variant of urothelial carcinoma in the literature it would prove difficult at the moment to be dogmatic regarding its biological behaviour and prognosis following treatment.

However, it would appear from the few reported cases of microcystic urothelial carcinoma that on the whole this tumour is a highly aggressive tumour.

Urologists, pathologists, and oncologists throughout the world should be encouraged to report new cases of microcystic urothelial carcinoma in order to document the biological behaviour of such a rare tumour.

## Figures and Tables

**Table 1:** A list of some of the reported cases of microcystic variant of urothelial carcinoma that have been reported in the literature with their outcome following treatment.

Authors	Sex/age	Site	Grade and stage	Treatment	Outcome	Followup duration
Leroy et al. 2002 [11]	M 73 years	Right renal pelvis	Lamina propria and muscularis invasion T2	Right nephrectomy	Died 18 months later from pulmonary metastasis	18 months
	F 62 years	Left renal pelvis	Tubules and cysts into lamina propria, muscularis propria, and renal parenchyma, and in renal pelvis to fat T3	Left nephroureterectomy	Alive after 6 months	6 months

Barresi et al. 2009 [9]	Details not available in our source of information	Urinary bladder	Metastatic with penile metastasis High-grade G3 invasive	Details not available in our source of information	Died of disseminated disease 6 months later	Six months

Alvarado-Cabrero et al. 2008 [10]	M 80 years	Urinary bladder	Superficial	Transurethral resection of the bladder tumour and intravesical mitomycin c	Alive and well after 3 years no recurrence	3 years

Vardar et al. 2007 [12]	M 52 years	Urinary bladder	Muscle-invasive	Radical cystectomy and pelvic lymph adenectomy	Alive at 10 months with no recurrence	10 months

Sari et al. 2007 [13]	M 56	Urinary bladder	High-grade (G3) Muscle-invasive with metastasis to penis	Radical cystoprostatectomy and total penectomy	He died 6 months postoperatively	6 months

Young and Zukerberg 1991 [5]	4 cases Age range 35 to 69 years	Urinary bladder	Deeply muscle invasive 4; Grade 2 2 cases Grade 3 2 cases	Details not available in source of information	Details not available in source of information	

Radopoulos et al. 2005 [15]	M 38 years	Urinary bladder	Muscle-invasive	Radical cystectomy	Alive 3 years with no recurrence	3 years

Pacchioni et al. 2009 [6]	M 56 years	Left renal pelvis	Details not available in source of information	Details not available	Details not available in source of information	
